# 

**Published:** 1903-12-19

**Authors:** 


### Publications.

Just Published. 8vo. with Photographic reproductions, 3/- net.

**THE PHYSIOGNOMY OF MENTAL DISEASES AND DEGENERACY.** By James Shaw, M.D., formerly Med. Supt. and Co-Licensee Haydock Lodge Asylum. Reprinted, with alterations and additions, from various articles in the "Medical Annual."

Just Published. Crown 8vo 1/6 net.

**A POCKET BOOK OF CLINICAL METHODS.** By Chas. H. Melland, M.D Lond., M.R.C.P., Phys. Ancoats Hosp.

THIRD EDITION. 12th Thousand. Small 8vo. Illustrated with 200 Original Drawings, 2/6. Lantern Slides of the Illustrations, 1/- and 1/6 each.

**"FIRST AID" TO THE INJURED AND SICK:** An Advanced Ambulance Handbook. By F. J. Warwick, B.A., M.B., and A. C. Tunstall, M.D., F.R.C.S.

THIRD EDITION. 2/- each. or 27/6 the Set of 16 Sheets, or mounted on linen, 45/- With nickel head.

**(Adopted by the War Office, Admiralty, and London School Board.)**

**LARGE SHEET "FIRST AID" DIAGRAMS:** Being Enlargements of the Illustrations in the above work on sheets 26 in. by 40 in., suitable for lectures and classes.

**Bristol: JOHN WRIGHT & CO.**

### MEDICAL ACCOUNTS.

Now is the time to arrange for this troublesome business, and

**WRIGHT'S THREE-BOOK SYSTEM,**

**WRIGHT'S SINGLE-BOOK SYSTEM,**

(11th Edition)

**WRIGHT'S VISITING LISTS, 1904,**

**WRIGHT'S CHARTS of all kinds,**

(Nearly 400,000 of which have been sold)

**WRIGHT'S CHART HOLDERS,**

(In use all over the world)

**WRIGHT'S CERTIFICATE BOOKS,**

**PROFESSIONAL ACCOUNT FORMS,**

**LETTER HEADINGS, and**

**DISPENSING LABELS.**

Are most of them better than the ordinary run of these things, and their use saves time and trouble.

PLEASE SEND FOR FREE SAMPLES.

**London: SIMPKIN & CO., Ltd.**

---

2nd Edition. Revised and Enlarged. Now Ready. Post 8vo. pp. xv.-258, and 39 Illustrations in the text. Cash price **4/6**, postage 4d.

### PRACTICAL TEXT-BOOK OF MIDWIFERY FOR NURSES.

By ROBERT JARDINE, M.D. Edin., M.R.C.S. Eng., F.F.P. and S Glas.

Professor of Midwifery in St. Mungo's College, Glasgow; Physician to the Glasgow Maternity Hospital; late President of the Glasgow Obstetrical and Gynæcological Society.

"It is the best suited for its purpose of any book of the kind which we have come across. . . . For nurses the little book is an improvement on any similar book of the kind with which we are familiar"
*The Dublin Journal of Medical Science.*

W. F. CLAY, 18 Teviot Place, Edinburgh.

Crown 8vo. Illustrated. Price **2s. 6d.** net.

### ANÆSTHETICS. By J. Blumfeld,

M.D.Cantab., Senior Anæsthetist to St. George's Hospital; Anæsthetist to the Grosvenor Hospital for Women and Children, London, and to the National Dental and Metropolitan Hospitals.

*Lancet.*—"....In writing this handbook the author has been quite successful." *Australasian Medical Gazette.*—"We can strongly commend this book." *Medical Chronicle.*—"The book is one which we can heartily recommend to senior students and practitioners."

London: Baillière, Tindall & Cox, 8 Henrietta St., Covent Garden.

Crown 8vo., price **3/6.**

With a "Plea for the State Registration of Nurses."

### MOCK NURSES OF THE LATEST FASHION.

By FREDERICK J. GANT, F.R.C.S., Consulting Surgeon to the Royal Free Hospital.

BAILLIÈRE, TINDALL & COX, Henrietta Street, Covent Garden, London.

JUST ISSUED. EIGHTH EDITION. Crown 8vo. **10s. 6d.**

### PHARMACY, MATERIA MEDICA AND THERAPEUTICS

is now rewritten and printed in large type, and is made a **companion to** the following volume. It contains an account of the action **of every drug in the B.P.**, as well as a section upon all the newest drugs, with an Introduction to the Art of Dispensing. With woodcuts, prescriptions, &c

Also FOURTH EDITION. 1055 pages, 8vo. **16s.**

### DICTIONARY OF TREATMENT.

Containing a Revised and up-to-date Article upon every Diseased Condition, whether Medical, Surgical, Gynæcological, or Ophthalmological, as well as Articles upon Poisoning, Drowning, and the various Surgical Emergencies and Accidents, arranged for Rapid Reference.

By W. WHITLA, M.A., M.D., Professor of Materia Medica, Queen's College, Belfast; Senior Physician to the Royal Victoria Hospital, &c. The Publisher will supply both volumes, carriage paid, for 21/-

London · HENRY RENSHAW 356 Strand.

Crown 8vo. cloth, price 5s. net; by post, 5s. 4d.

*Second Edition of*

DR. HEYWOOD SMITH'S

### PRACTICAL GYNÆCOLOGY.

This is a genuine handbook on the subject, intended for ready reference by busy practitioners.

W. J. GLAISHER, 57 Wigmore Street, Cavendish Square, W.

JUST OUT. With 3 coloured Plates, and 54 other Illustrations. Price 6/- net.

### HANDBOOK OF DISEASES OF THE EAR

For Students and Practitioners.

By RICHARD LAKE, F.R.C.S. Eng., Surgeon to the Royal Ear Hospital.

London: BAILLIÈRE, TINDALL & COX, 8 Henrietta Street, Covent Garden.

### IMPORTANT NOTICE.

**On account of the Christmas Holidays, we beg to announce that we shall go to press with our issue of the 26th DECEMBER on MONDAY NIGHT, the 21st inst. Advertisements intended for insertion in this issue should therefore reach us not later than 4 p.m. ON MONDAY, the 21st inst.**

### NOTICE TO CORRESPONDENTS.

All MSS., Letters, Books for Review, and other matters intended for the Editor should be addressed THE EDITOR, "THE HOSPITAL," THE HOSPITAL BUILDINGS, 28 & 29 SOUTHAMPTON STREET, STRAND, W.C.

The Editor cannot undertake to return rejected MS., even when accompanied by stamped directed envelope.

**Notice to Advertisers and Subscribers.**

All Advertisements, Orders for Copies of THE HOSPITAL, and Business Communications should be addressed to **THE MANAGER (Not to the Editor)**, THE HOSPITAL, 28 & 29 Southampton Street, Strand, London, W.C.

The Cost of Subscription, post free, is as follows:—Per week, 3½d.; per quarter, 3s. 9d.; per half-year, 7s. 6d.; per annum, 15s. Foreign and Colonial:—Per annum, 19s. 6d., payable, in all cases, in advance.

**NOTICE.—Vol. XXXIV. of "The Hospital" (April 1903 to September 1903), in handsome cloth binding, is now on sale at the office of this Journal, price 10s. 6d. Cases for binding the Numbers contained in this Volume 2s. Index to Vol. XXXIV., 2½d., post free.**

**The Monthly part for December is now ready. Price 1s., by post, 1s. 3d.**

### BOOKS RECEIVED.

Baillière, Tindall and Cox.

"Manual of Massage." Second edition. By M. A. Ellison.

"Nutrition of the Infant." By Ralph Vincent.

Macmillan and Co.

"Archives of the Middlesex Hospital." Vol. I.

Longmans and Co.

"The Prevention of Consumption." By A. Hillier.

Charles Griffin and Co.

"Official Year-book of Scientific and Learned Societies."

Rebman, Ltd.

"Practice of Obstetrics." By J. C. Edgar.

The Scientific Press.

"Examination of the Urine." By J. K. Watson, M.D.

"Treatment of Plague with Professor Lustig's Serum." By N. H. Choksy.

"Hints on How to Start a District Nursing Association." By a County Superintendent.

Messrs. J. and A. Churchill.

"English Handbook to the Paris Medical School." By A. A. Warden, M.D.

Midland Educational Company.

"Hope in Shadow Land." By T. J. Bass.

### THE STUDENT OF MEDICINE.

### APPOINTMENTS.

**J. M. Acland**, M.R.C.S., L.D.S.Eng., Dental Surgeon to the Royal Devon and Exeter Hospital. **Henry Andrew**, M.R.C.S. Eng., L.R.C.P.Lond., Honorary Anæsthetist to the Royal Devon and Exeter Hospital, and Anæsthetist to the Devon and Exeter Dental Hospital. **George W. Badgerow**, M.B.Tor., L.R.C.P.Lond., M.R.C.S.Eng., House Surgeon to the Throat Hospital, Golden Square. **W. Langworthy Baker**, M.R.C.S.Eng., L.R.C.P.Lond., Assistant Resident Medical Officer at the Toxteth Union Workhouse. **Jas. B. Bird**, M.D.Edin., Physician to the Cumberland Infirmary. **H. W. Bruce**, M.D., Assistant Medical Superintendent of the Southwark Union Infirmary. **F. A. Coward**, L.R.C.P.&S.Edin., District Medical Officer of the Cannock Union. **Edward Davis**, L.D.S.Eng., Honorary Surgeon-Dentist to the Queen Adelaide's Dispensary, Pollard Row, Bethnal Green, E. **P. J. De Nyssen**, M.R.C.S., L.R.C.P., Certifying Factory Surgeon for the Halesworth District, Suffolk. **Brennan Dyball**, M.B., B.S.Lond., F.R.C.S.Eng., Honorary Anæsthetist to the Royal Devon and Exeter Hospital, and Assistant Anæsthetist to the Devon and Exeter Dental Hospital. **Arnold Edwards**, M.D. Vict., Ch.B., Honorary Assistant Surgeon to the Manchester and Salford Lock Hospital. **J. Graham Forbes**, M.D., D.P.H.Cantab., Clinical Pathologist and Bacteriologist to the Hospital for Sick Children, Great Ormond Street, W.C. **W. L. Garner**, M.B., B.C.Cantab., District Medical Officer of the Amphill Union. **W. Conway Gent**, L.R.C.P., L.R.C.S.Edin., Medical Officer No. 2 District, Faringdon Union. **J. A. K. Griffiths**, M.B.Lond., M.R.C.S., District Medical Officer of the Knighton Union. **P. J. Hamilton**, M.B., B.Ch., R.U.I., Certifying Factory Surgeon for the Arklow District, Wicklow. **J. O. Jones**, L.R.C.P.&S.Edin., District Medical Officer of the Holywell Union. **C. Royds Jones**, M.B., Ch.B.Vict., Honorary Surgeon to the Royal South London Dispensary. **A. W. J. Macfadden**, M.B., C.M.Edin., D.P.H. Camb., Medical Officer of Health of the Edmonton Urban District. **P. de C. Potter**, M.R.C.S.Eng., L.S.A., District Medical Officer of the Leicester Union. **J. Lloyd Roberts**, M.D., B.S., B.Sc., B.A.Lond., M.R.C.P.Lond., F.R.C.S.Eng., Honorary Physician to the Royal Southern Hospital, Liverpool. **James H. Sequeira**, M.D.Lond., M.R.C.P.Lond., F.R.C.S.Eng., Physician in charge of the Skin Department of the London Hospital. **Reginald V. Solly**, M.D.Lond., F.R.C.S.Eng., Medical Registrar and Pathologist to the Royal Devon and Exeter Hospital. **Ethel Miller Vernon**, M.D., B.S.Lond., Attending Medical Officer, Western Dispensary, Rochester Row, Westminster. **T. A. Walker**, L.R.C.P. Edin., M.R.C.S.Eng., Certifying Factory Surgeon for the Weymouth District, Dorsetshire.

### "The Hospital" Institutional Library.

"Hospitals and Asylums of the World." 4 Vols., with a Portfolio of Plans, complete £12 12s.

"Burdett's Hospitals and Charities, 1903." 5s. net.

"Burdett's Official Nursing Directory, 1903." 3s. net.

"Cottage Hospitals, General, Fever, and Convalescent." 10s. 6d.

"Uniform System of Accounts for Hospitals and Public Institutions." New Edition. 4s. net.

"Hospital Expenditure: The Commissariat." 2s. 6d.

"A Legal Handbook for the Use of Hospital Authorities." 2s. 6d.

"The Cottage Hospital Case Book or Register of Patients." 10s. and 12s. 6d.

All these are published by the Scientific Press, Ltd., and may be obtained through any bookseller, or direct from the publishers, 28 and 29 Southampton Street, Strand, London, W.C.

THE NURSING PROFESSION: How and Where to Train.

By Sir HENRY BURDETT, K.C.B.

A reliable and up-to-date Guide to Training for the calling of a Nurse. In its pages would-be Nurses will find all the information they require.

New Edition. Price 2/-, post free 2/4.

Of all Booksellers or of THE SCIENTIFIC PRESS, LTD., 28 & 29 Southampton Street, Strand, London, W.C.

NURSES.

1/6 *Post Free.*

For valuable information on Invalid Cookery and a list of Dainty and Appetising Recipes for Invalids, Dyspeptics, and Convalescents,

1/6 *Post Free.*

YOU SHOULD OBTAIN

THE ART OF FEEDING THE INVALID.

By a MEDICAL PRACTITIONER and a LADY PROFESSOR OF COOKERY.

Over 250 pp., bound in Cloth Boards, 1s. 6d. post free.

THE SCIENTIFIC PRESS, LTD., 28 & 29 Southampton Street, Strand, London, W.C.

*Just Published.*

### THE EXAMINATION OF THE URINE.

By J. K. WATSON, M.D.Edin., M.B., C.M.,

*Author of "A Handbook for Nurses."*

Price 1/- net; post free 1/1.

*Of all Booksellers, or of*

THE SCIENTIFIC PRESS, LIMITED,

28 & 29 Southampton Street, Strand, London, W.C.

### USEFUL XMAS PRESENTS.

### AN IDEAL GIFT.

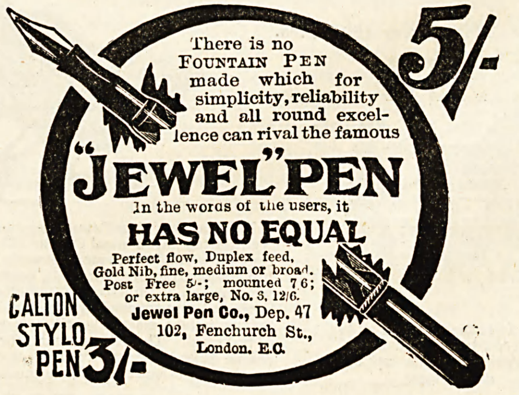

### J. & A. CHURCHILL'S

*Books for Nurses.*

### Suitable Presents For CHRISTMAS and the NEW YEAR

### NURSING.
Nursing, General, Medical and Surgical, with an Appendix on Sick Room Cookery. By Wilfred J. Hadley, M.D., F.R.C.P., F.R.C.S., Physician and Pathologist to the London Hospital, Lecturer to Nurses at the London Hospital Nursing School . . . . 3s. 6d.

### MEDICINE.
Lectures on Medicine to Nurses. By Herbert E. Cuff, M.D., F.R.C.S., Medical Superintendent, North Eastern Fever Hospital London. Fourth Edition. With 29 Illustrations . . . . . 3s. 6d.

### INFANT FEEDING.
The Natural and Artificial Methods of Feeding Infants and Young Children. By Edmund Cautley, M.D., F.R.C.P., Physician to the Belgrave Hospital for Children; late Casualty Physician and Assistant Demonstrator of Physiology, St. Bartholomew's Hospital. Second Edition. 7s. 6d.

### DIET.
Diet and Food considered in relation to Strength and Power of Endurance, Training and Athletics. By A. Haig, M.D., F.R.C.P. Fourth Edition. 2s.

### MIDWIFERY.
A Short Practice of Midwifery for Nurses. By Henry Jellett, M.D. (Dub. Univ.), F.R.C.P.I., Ex-Assistant Master, Rotunda Hospital, Dublin, Extra Examiner in Midwifery and Gynæcology, Royal College of Physicians of Ireland, Extern Examiner in Midwifery, Royal University of Ireland. With 67 Illustrations . . . . 6s.

### MONTHLY NURSING.
A Short Manual for Monthly Nurses. By C. J. Cullingworth, M.D., F.R.C.P., Obstetric Physician to St. Thomas's Hospital. Revised with the assistance of M. A. Atkinson, Matron of the General Lying-in Hospital, London. Fifth Edition. 1s. 6d.

### HEALTH OF A WIFE.
Chavasse's Advice to a Wife on the Management of her Own Health. Fourteenth Edition. By Fancourt Barnes, M.D., Consulting Physician to the British Lying-in Hospital . . . . 2s. 6d.

### HEALTH OF CHILDREN.
Chavasse's Advice to a Mother on the Management of her Children. Fifteenth Edition. By George Carpenter, M.D., Physician to the Evelina Hospital for Children . . . . . . 2s. 6d.

### ANTISEPTICS.
Lessons in Disinfection and Sterilisation; an Elementary Course of Bacteriology, with a Scheme of Practical Experiments. By F. W. Andrewes, M.D., F.R.C.P., Lecturer on Pathology at St. Bartholomew's Hospital. With Illustrations . 3s. net. [*Just Published.*

### SURGERY.

A Manual of Minor Surgery and Bandaging. By Christopher Heath, F.R.C.S., and Bilton Pollard, F.R.C.S., Surgeon to University College Hospital. Twelfth Edition. With 195 Illustrations. 6s. 6d.

### HYGIENE.
The Elements of Health: An Introduction to the Study of Hygiene. By Louis C. Parkes, M.D., D.P.H.Lond., Lecturer on Public Health at St. George's Hospital. 3s. 6d.

### DICTIONARY.
A Short Dictionary of Medical Terms. By R. G. Mayne M.D., LL.D. . 2s. 6d.

7 GREAT MARLBOROUGH STREET, LONDON.

